# COVID-19 pandemic has disrupted the continuity of care for chronic patients: evidence from a cross-sectional retrospective study in a developing country

**DOI:** 10.1186/s12875-023-02086-6

**Published:** 2023-07-01

**Authors:** Abbasali Dehghani Tafti, Azadeh Fatehpanah, Ibrahim Salmani, Mohammad Amin Bahrami, Hossien Tavangar, Hossien Fallahzadeh, Ali Ahmadi Tehrani, Sajjad Bahariniya, Gholamreza Ahmadi Tehrani

**Affiliations:** 1 Department of Health in Disater and Emergencies, School of Public Health, Shahid Sadoughi University of Medical Sciences, Yazd, Iran; 2 Healthcare Management Department, School of Health Management and Information Sciences, Shiraz University of Medical Sciences, Shiraz, Iran; 3 School of Nursing and Midwifery, Nursing and Midwifery Care Research Center, Shahid Sadoughi University of Medical Science, Yazd, Iran; 4 Center for Healthcare Data Modeling, Departments of Biostatistics and Epidemiology, School of Public Health, Shahid Sadoughi University of Medical Sciences, Yazd, Iran; 5 Pharmaceutical Research Center, Shahid Sadoughi University of Medical Sciences, Yazd, Iran; 6 Health Services Management Department, School of Public Health, Shahid Sadoughi University of Medical Sciences, Yazd, Iran; 7 International Campus, Shahid Sadoughi University of Medical Sciences, Yazd, Iran

**Keywords:** Continuity of Care, Hypertension, Diabetes, Chronic care, COVID-19

## Abstract

**Background:**

Any disruption in continuity of care for patients with chronic conditions can lead to poor outcomes for the patients as well as great damage for the community and the health system. This study aims to determine the continuity of care for patients with chronic conditions such as hypertension and diabetes during COVID-19 pandemic.

**Methods:**

Through a cross-sectional retrospective study, data registered in six health centers in Yazd, Iran were analyzed. Data included the number of patients with chronic conditions (hypertension and diabetes) and average daily admission during a year before COVID-19 pandemic and the similar period after COVID-19 outbreak. The experience of continuity of care was assessed applying a validated questionnaire from a sample of 198 patients. Data analysis was done using SPSS version 25. Descriptive statistics, independent T-Test and Multivariable regression were used for analysis.

**Findings:**

Results indicate that both visit load of the patients with chronic conditions (hypertension and diabetes) and their average daily admission were decreased significantly during a year after COVID-19 pandemic compared to the similar period before COVID-19 outbreak. The moderate average score of the patients` experience towards continuity of care during the pandemic was also reported. Regression analysis showed that age for the diabetes patients and insurance status for the hypertension patients affect the COC mean scores.

**Conclusion:**

COVID-19 pandemic causes serious decline in the continuity of care for patients with chronic conditions. Such a deterioration not only can lead to make these patients` condition worse in a long-term period but also it can make irreparable damages to the whole community and the health system. To make the health systems resilient particularly in disasters, serious attention should be taken into consideration among them, developing the tele-health technologies, improving the primary health care capacity, designing the applied responsive models of continuity of care, making multilateral participations and inter-sectoral collaborations, allocating sustainable resources, and enabling the patients with selfcare skills are more highlighted.

## Background

Increasing life expectancy and as a result the population aging have led to an increase in the rate of chronic disease such as diabetes mellitus (DM) and hypertension (HT( [1]. Management of chronic non-communicable diseases (NCDs) is typically long term and requires ongoing health- care interventions [2]. Integrated people-centered health services frameworks suggest the practice of continuity of care in primary healthcare to improve the management on NCDs [2]. Multidisciplinary treatment and continuous care throughout treatment are important for ensuring disease control and avoiding complications in NCDs such as DM [1]. Continuity of Care (COC) is defined as the extent to which healthcare services are received in a coordinated and uninterrupted manner by the patients [3]. Evidence demonstrates that COC is strongly associated with patients’ and health system’s outcomes such as improved health status; patient satisfaction and quality of life; reduced hospitalization rates; reduced mortality rates; improved patients’ self-efficacy and adherence to treatment and reduction of health expenditures [4–15]. Chan et al. (2021) in a systematic review and meta-analysis of current literature reported a strong association between higher continuity of care and reduced mortality rate, complication risks and health service utilization among DM and/or HT patients [2]. Cho et al. (2015) in a nationwide study in Korea have reported the same findings [16]. In addition there is evidence which shows that COC and continuous communication with healthcare providers also improves patients’ self-management skills, which in turn contributes to better disease control [17, 18].

Despite this importance of care continuity for chronic patients, reports and evidence show that the COC for chronic diseases has significantly been disrupted during the COVID-19 pandemic which can lead to negative outcomes for patients; communities and health systems [19–25].

For example, findings of WHO`s opinion assessment from 155 members have shown that during the first wave of the pandemic, a major decline was occurred in delivering health care services and continuity of care for non-communicative disease. Almost half of the studied countries have reported that their routine care for patients suffering from hypertension decreased [23–25].

According to what was considered above, COVID-19 pandemic and the experience of decline in continuity of care, especially when supposed an increase in the rate of chronic diseases now more than ever emphasize the necessity of health systems readiness and enabling the community for continuity of care during health disasters. Therefore, this study was conducted with the aim of determining the continuity of care for patients with chronic conditions such as hypertension and diabetes during COVID-19 pandemic in a developing country. It is notable that the aim of this study was not to analyze patients’ clinical parameters. This work was aimed to investigate the status of continuity of care for hypertension and diabetes patients during the pandemic. In this study, using data from the health system as well as those collected from patients, we examined the continuity of chronic patients’ care during crisis in order to sensitize policy makers about the issue and find solutions to ensure continuity of care for chronic patients in similar conditions. In fact, the focus of this study was on continuity of care with the aim of strengthening the health system to face health crises. Therefore, clinical data were not analyzed in this study. Also, we selected DM and HT for study considering that these conditions are among the most common chronic conditions in our country as well as the other societies. Also, DM and HT, which are the lifelong conditions and need the careful management, increase the risk of developing other conditions such as heart diseases. Indeed, we chose these two diseases because the main focus of this study was primary care. In our country, like many others, routine care of DM and HT is provided at the primary care level while other chronic patients, such as cancer patients, receive the majority of their routine care from specialized hospitals.

## Methods

### Study design

It was a retrospective longitudinal and multilevel survey applying two sources of data including the registered data from six health centers in Yazd, Iran, and the self-assessment of the patients about the continuity of care for patients with chronic conditions (hypertension and diabetes) during COVID-19 pandemic.

### Study population

The study population consisted of all those patients suffering from type 2 diabetes and hypertension who had an active medical record in one of the Yazd`s health centers from at least two years before COVID-19 pandemic. The inclusion criteria consist of those patients with at least 18 years old and strong tendency to participate in the study. Considering this, all the patients with active medical records were included for the first phase of the study.

For the second phase, the questionnaire survey, a two-level cluster sampling was applied based on the health center and availability of the background non-communicable disease (hypertension or diabetes). For this purpose, six health centers were selected randomly and then 33 patients suffering from diabetes and 33 patients with hypertension were included applying an accidental sampling.

### Data collection process and instrument

At the first phase, demographic, and clinical information of the participants including some variables like age, gender, marital status, level of education, occupation, residency, type of insurance, type of disease and history of their health were collected. Data relevant to the patients` average rate of visits and average daily admission were also collected in this phase applying the databases of the health centers and the patients` self-assessment. For this purpose, firstly, data registered via patients` medical records including their demographic information and the average rate of visits to the health centers were extracted for the first year after COVID-19 pandemic and the similar period before the outbreak. Then, the number of patients` visits for those patients suffering from diabetes and hypertension during COVID-19 pandemic were extracted from th0eir self-reports.

At the second phase, the patients` experience from continuity of care was assessed applying a standard moderated questionnaire of Gulliford [9]. This Patient Continuity of Care Questionnaire (PCCQ) contains 19 questions in four dimensions as follows: Longitudinal continuity, Flexible continuity, Relational continuity and Team and cross-boundary continuity. The participants supposed to answer to each question in a spectrum from “never” to “five times or more”, “In five days or more” to “at the same day”, from “very low” to “excellent”, “extremely good” to “extremely bad”, from “extremely difficult” to “extremely easy” and from “completely agree” to “completely disagree”.

According to the questionnaire manual, the total score of the questions was calculated between 0 and 95 (Longitudinal continuity = 20, Flexible continuity = 20, Relational continuity = 25 and Team and cross-boundary continuity = 30). Such a score scale was divided into three levels of low (0–31), moderate (32 to 63) and high (64 to 95).

The questionnaire was validated in Persian language prior to the study. The validation process was done as follows: at the first, the original questionnaire was translated into Farsi using the forward-backward method. The initial translation from the original language was done by two independent translators fluent in both the English and Farsi. Discrepancies between the two translators were discussed and resolved between the original translators with the addition of new translator who was not involved in the initial translations. Then, the initial translation was independently back-translated to ensure the accuracy of the translation. In the next step, the translated version of questionnaire was discussed in an expert committee consisting of the translators, research team and five experts who were familiar with the subject. In this committee the discrepancies were solved and a consensus was reached on the all items. Then the pre-final translated version was pilot tested on a 24 patients of the study population. The participants of this pilot study were selected from all the studied health centers. In this pilot study, the respondents were verbally asked to explain what they thought each questionnaire item and their corresponding responses meant. Gathered data from pilot study were analyzed and necessary corrections were made. This pilot study allowed the researchers to make sure that the translated items retained the same meaning as the original ones and to ensure that there is no confusion regarding the translated questionnaire. In the next step, the content validity of the final translated version was qualitatively assessed in an expert panel consisted of research team and 12 experts from the studied health centers. In this panel the content validity of the entire questionnaire was approved. In the last step, reliability of the questionnaire was evaluated using Cronbach’s alpha. For this, the questionnaire was completed by 30 patients from the study environment and Cronbach’s alpha was measured for the entire questionnaire as 0.90 and between 0.87 and 0.94 for dimensions.

### Data analysis

Data was analyzed applying SPSS software version 25. Descriptive statistics were used as well as independent T-Test and multivariable regression.

### Ethical considerations

This study was conducted according to the international protocols of research ethics. All participants provided a written informed consent. All participants provided a written informed consent. For illiterate participants, the text of the informed consent was read for them orally by the researchers and then their consent was obtained. Also, any explanation they needed was provided to them. All the study procedures were approved by the Research Ethics Committee affiliated with Yazd Shahid Sadoughi University of Medical Sciences with the ID of: IR.SSU.SPH.REC.1400.187.

## Results

The descriptive and demographic characteristics of the participants are presented in Table 1. According to Table 1, the most frequent patients both with diabetes and hypertension belong to the age group of 50–60 years old. 54.5% of those studied participants who were suffering from diabetes and 58.1% of those suffering from hypertension were female respectively while the rest of them were male. Other descriptive results show that the majority of the participants in both groups were married and benefited from public and social security insurance. Considering the education level, most of the participants graduated from junior school.

**Table 1:** Demographic characteristics of the participants

Variables		Patients suffering from diabetes (N/%)	Patients suffering from hypertension (N/%)
**Age**	30–40	1 (0.5%)	1 (0.5%)
	40–50	36 (18.2%)	29 (14.6%)
	50–60	101 (51%)	84 (42.4%)
	60–70	46 (23.2%)	59 (29.8%)
	70–80	14 (7.1%)	25 (12.6%)
**Gender**	Female	108 (54.5%)	115 (58.1%)
	Male	90 (45.5%)	83 (41.9%)
**Marital Status**	Married	166 (83.8%)	168 (84.8%)
	Divorced	3 (1.5%)	1 (0.5%)
	Widow/er	29 (14.6%)	29 (14.6%)
**Educational status**	Illiterate	52 (26.3%)	60 (30.3%)
	Primary school	36 (18.2%)	28 (14.1%)
	Junior school	75 (37.9%)	60 (30.3%)
	High school	31 (45.7%)	44 (22.2%)
	Graduate certificate and Bachelor	4 (2%)	6 (3%)
**Occupation status**	Employed	54 (27.3%)	40 (20.2%)
	Retired	42 (21.2%)	43 (21.7%)
	Housekeeper	102 (51.5%)	115 (58.1%)
**Insurance status**	Social security	144 (72.7%)	137 (69.2%)
	Health insurance	54 (27.3%)	57 (28.8%)
	Others	0	1 (0.5%)
	No insurance	0	3 (1.5%)

**Fig. 1:**
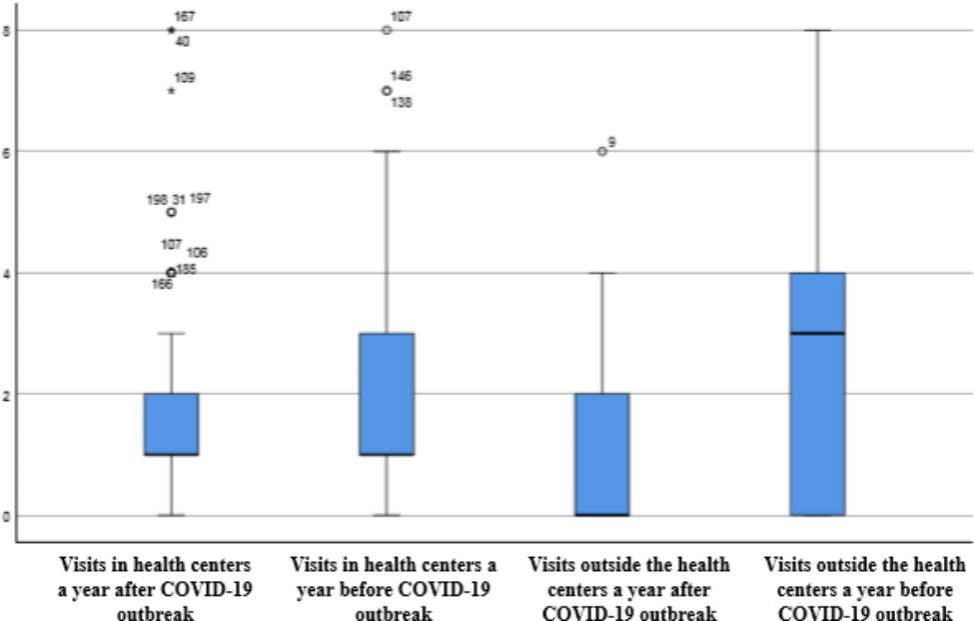
The mean of diabetes patients’ visits in health centers and outside it a year before and after COVID-19 pandemic

**Fig. 2:**
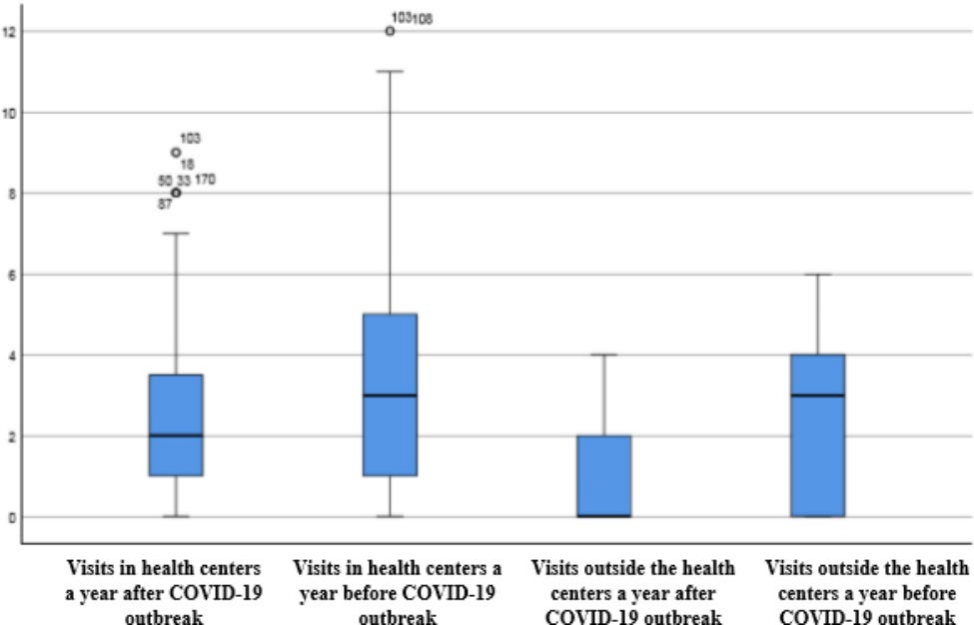
The mean of hypertension patients’ visits in health centers and outside it a year before and after COVID-19 pandemic

Table 2; Figs. 1 and 2, compare the average rate of visit for each of the patients’ groups suffering from diabetes or hypertension during a year before and after COVID-19 pandemic. The average rate of visits for patients suffering from diabetes or hypertension was higher in a year before COVID-19 pandemic compared to the similar period after COVID-19.This table shows that the number of visits of diabetes and hypertension patients, both in health centers and outside of it, in the year after the outbreak of the pandemic has significantly decreased compared to the year before the outbreak (P < 0.05).

**Table 2:** The average of visits to health centers by patients with diabetes and hypertension a year before and after COVID-19 pandemic

Number of visits	Diabetes				Hypertension			
	N	Mean	SD	P_value_^*^	N	Mean	SD	Pvalue^*^
Number of visits in the health center a year after COVID-19	197	1.47	1.38	0/005^**^	176	2.50	2.04	0/000^**^
Number of visits in the health center a year before COVID-19	197	1.83	1.71		176	3.36	2.80	
Number of visits outside the health center a year after COVID-19	198	0.73	1.05	0/000^**^	198	0.86	1.06	0/000^**^
Number of visits outside the health center a year before COVID-19	198	2.46	2.20		198	2.69	2.10	

^*^ **Independent T-Test**

^**^ **Significant at P < 0/05**

Other results illustrated in Table 3, demonstrate the average daily admission of the patients for each of the diabetes or hypertension groups. According to Table 3, the average daily admission of the patients in both groups was higher among all the studied health centers in a year before COVID-19 pandemic compared to the similar period after COVID-19. The only exception was related to the average daily admission for those patients suffering from hypertension in the 4th health center which illustrated an incremental trend after COVID-19 pandemic. Follow up findings indicate that this increase was mainly related to the tele-consult which was implemented in the 4th center during the pandemic. Table 4 presents the mean score of the continuity of care from the participants` viewpoint. In both studied groups, longitudinal continuity had the lowest score while relational had the highest score. Comparing the total score of continuity of the care, the score was determined in a moderate level for those patients suffering from diabetes but categorized at the moderate to good level boarder for the second group (Those suffering from hypertension). Also, as shown in this table the mean scores of two studied groups (diabetes vs. hypertension) is significantly different in relational continuity but the means of other dimensions and total score of two groups have no significant differences. The results of multivariable regression analysis to investigate predictors of COC mean scores are presented in Tables 5 and 6. Age, gender, education and insurance status were included in the regression analysis. As shown in Tables 5 and 6, only the age for the diabetes patients and insurance status for the hypertension patients affect the COC mean scores.

**Table 3:** The average of patients daily admissions to health centers by patients with diabetes and hypertension a year before and after COVID-19 pandemic

Visits in health centers	Diabetes				Hypertension			
	Number of visits a year after pandemic	Average of admission a year after pandemic	Number of visits a year before pandemic	Average of admission a year before pandemic	Number of visits a year after pandemic	Average of admission a year after pandemic	Number of visits a year before pandemic	Average of admission a year before pandemic
**Health Center 1**	482	1.65	708	2.44	730	2.50	1475	5.04
**Health Center 2**	299	1.02	380	1.31	446	1.53	1035	3.58
**Health Center 3**	174	0.59	389	1.34	375	1.28	1209	4.18
**Health Center 4**	239	0.82	348	1.20	1336	4.59	1012	3.50
**Health Center 5**	185	0.63	223	0.77	322	1.10	437	1.51
**Health Center 6**	17	0.05	93	0.32	175	0.60	545	1.88
**Total**	1396	4.79	2141	7.40	3384	11.62	5695	19.70

**Table 4:** The mean score of COC among the patients with diabetes and hypertension during COVID-19 pandemic

Variable	Diabetes			Hypertension			P_value_^*^
	No.	Mean	SD	No.	Mean	SD	
Longitudinal continuity	198	7.39	3.63	198	7.70	3.86	0/244
Flexible continuity	198	15.57	1.76	198	15.57	1.74	0/886
Relational continuity	198	19.83	2.48	198	23.84	3.00	0/017^**^
Team and cross-boundary continuity	198	16.54	2.42	198	16.20	2.18	0/260
Total	198	59.35	6.20	198	63.32	6.22	0/698

^*^ **Independent T-Test**

^**^ **Significant at P < 0/05**

**Table 5:** The regression analysis of COC Mean scores’ predictors for diabetes patients

	Unstandardized Coefficients		Standardized Coefficients Beta	t	Sig.
	B	Std. Error			
Constant	49.675	3.638	-	13.654	0.000
Age	-1.164	0.860	-0.093	-1.354	0.000
Gender	-0.639	1.017	-0.046	0.628	0.177
Insurance Status	2.518	0.676	0.336	3.722	0.531
Education	-0.043	0.482	-0.008	-0.090	0/928

**Table 6:** The regression analysis of COC Mean scores’ predictors for Hypertension patients

	Unstandardized Coefficients		Standardized Coefficients Beta	t	Sig.
	B	Std. Error			
Constant	60.389	4.283	-	14.098	0.000
Age	1.199	0.688	0.168	1.743	0.083
Gender	1.245	1.011	0.093	1.232	0.220
Insurance Status	-2.772	1.204	-0.193	-2.032	0.023
Education	-0.763	0.530	-0.139	-1.438	0/152

## Discussion

Global reports indicate a serious restriction in the patients with chronic conditions` access to routine health care services during the outbreak [26–30]. With this perspective, conducting a study to determine the access level of those patients with non-communicable diseases during the pandemic could be handy and useful for health systems and health policy makers. So, this study was conducted with the aim of determining the continuity of care for patients with hypertension and diabetes during COVID-19 pandemic in Yazd, Iran.

Our results imply that the rate of visits for both groups of the patients who were suffering from the hypertension and diabetes, both in health centers and outside them, and the average rate of the health centers’ daily admissions for these patients were significantly lower a year after COVID-19 pandemic comparing with the similar period before the pandemic. Although, a little difference in the reduction rate of visits for diabetes and hypertension patients was reported but this difference was not significant. This difference between 2 groups of patients can be caused by the nature of diseases and the different care plan for each one at health centers. Also, our findings showed that the decrease in patients’ visits outside the health centers was considerably higher than the decrease in visits in the health centers for both groups of patients.

Overall, our results approve the considerable disruption of COC for diabetes and hypertension patients during the pandemic. Such a restriction firstly occurred due to the concentration of the health systems on managing the pandemic during a long period. Other reasons for this restriction can be because of decrease in access to health care specialists, health care workers` burnout and the economic challenges at the macro level [26–30]. Quarantine and travel restrictions as same as the fear of diabetes and hypertension patients from getting infected with Corona are the other factors that limited the access of patients to routine care during pandemic.

Serious disruption in the access of chronic patients to routine care has been evidenced and reported from other countries, as same as our findings. Comparing the present results with the available findings from other studies reveals some similarities despite the differences among the studied populations. For instance, according to the results of a time-series study conducted by Doubova et al. (2022) in Mexico, about 8.74 million visits were cancelled among the nine health services studied from the related data of the national Mexican Social Security which covers almost half of the local population. Specifically considering these results, patients suffering diabetes lost about one third of their routine care during COVID-19 pandemic. Such a decline led to a 22% decrease in the rate of those patients whose diabetes status was under control. According to Doubova et al. (2022), COVID-19 pandemic has affected the resilience of Mexican health system to a great extent particularly in the area of providing necessary health care services [31]. Another study by Hoffman (2022) has demonstrated the prevention status of chronic non-communicable diseases in the USA. Based on the results of this study, COVID-19 has acted as a factor to reveal the available declines of the global public health systems [32]. Similarly, Balasuriya et al. (2022), emphasized the significant role of COVID-19 pandemic as a factor which shows the necessity of preventing chronic disease more than ever. Regarding Balasuriya et al. (2022), COVID-19 has played a significant role as an inhibitor towards appropriate access to health care services. The pandemic caused a decline in receiving on time health care as well as socioeconomic challenges and behavioral changes [33]. Like the present results in Yazd, Iran, Kendzerska et al. (2021), in their study have reported a significant decrease in the number of in-person and elective visits for patients with chronic conditions due to the governmental restriction during the pandemic [34]. Danhieux et al. (2020) also considered the primary health care during COVID-19 in Belgium from the health care providers` perspective. According to their findings, COVID-19 was considered as a reason of decline in chronic care and continuity of primary health care in Belgium [35]. Chudasama et al. (2020), in a global opinion assessment from health care experts in 47 countries have concluded that during the pandemic, chronic patients` access was affected seriously. As a result, they have strongly recommended the continuity of care for chronic patients as a determinant factor to prevent side effects and mortalities non-related to COVID-19 [36]. Another study designed by Gadsden et al. (2022) in Southeast Asia announced that COVID-19 has declined the delivery of necessary health care services particularly for non-communicable diseases. As they mentioned, public preventive actions like national quarantine and traffic restrictions which were normally applied as a response to pandemic control, were among the main reasons of decreasing the patients` access to their routine care [37]. Resembling the present results, Statchels et al. (2022) have reported that from the beginning of the pandemic, many of the regular and routine medical visits were postponed, delayed, or cancelled due to some reasons like fear, lack of tendency, isolation, lack of health care workers, and problems in primary health care delivery [38]. As this study was concentrated on the status of continuity of care during COVID-19 pandemic in a developing country, results of Ayele Ta et al. (2022) in northeast of Ethiopia were also confirmed the effects of COVID-19 pandemic on the patients` health care seeking behaviors. According to their findings, in a developing country with the restricted resources, the condition can be worsened and significant loss of visits and increase in the number of postponed, cancelled, and delayed visits can be intensified mainly due to higher frequency and prevalence of positive or suspected cases of COVID-19, higher rate of out-of-pocket payments and lack of medical record systems [39]. Other studies such as Sumner et al. (2022), Javanparast et al. (2021), Wang. (2021), Cuschieri, and Mamo (2021), Sakur et al. (2022), Moore (2022), Hacker et al. (2021) and Burayzat et al. (2022) also reported similar findings from the negative effects of the pandemic on the COC for chronic patients and emphasized the necessity of appropriate actions by health systems to ensure the routine care of these patients during health disasters [40–48].

In this study we analyzed a questionnaire data of patients’ experience from COC during pandemic in addition to the health system’s data. For this purpose we applied the multidimensional model of COC, which Gulliford et al. have developed. In this model, COC has been defined as a multidimensional concept that has 4 dimensions including Longitudinal Continuity (LC); Flexible continuity (FC); Relational Continuity (RC), and Team and Cross-boundary Continuity (TCB). Longitudinal continuity has been defined as the regular monitoring of a patient’s condition over the time. This monitoring should be accompanied by counseling on self-care and self-management and should be performed by as few clinicians as possible. Longitudinal continuity of care requires the development of a relationship based on familiarity, closeness and honesty between the patient and the care team. This relationship creates another dimension of continuity of care, which is called relational continuity. Chronically ill patients may sometimes need urgent consultation with their care providers due to emergency problems related to their illness. Flexible continuity refers to the extent to which care providers flexibly respond to these changing needs of patients in a timely manner. Caring for chronic patients requires a great deal of coordination and cooperation among settings and professionals who provide it. The last dimension of COC that is named experienced team and cross boundary continuity refers to the degree of coordination and consistency of care between these settings and professionals in providing care [9]. In this regards, our findings indicated that the experience of the studied patients about continuity of their routine care during the pandemic was not appropriate and acceptable. Based on the findings, the participated patients rated their experiences from the COC and its dimensions including Longitudinal; Flexible; Relational and Team and Cross-boundary continuity as moderate. In both groups of patients, relational continuity got the highest mean while longitudinal continuity got the lowest mean. HT patients rated their relational continuity significantly better than DM patients but two groups had no difference in the other dimensions as same as the total COC mean. Although we have no records of our participants’ experience before the pandemic for the comparison but there is some literature to discuss this. Gulliford et al. (2006), in their study of type 2 patients in England have reported the mean score of experienced continuity of care for diabetes mellitus (ECC-DM) as 62.1 (ranged from 0 to 100). Also, they have reported that the mean of ECC-DM varied significantly between practices, ranging from 46 to 78 at different family practices. Experienced continuity was lower for patients receiving only hospital clinic care than for those receiving some diabetes care from their family practice. Patients had higher ECC-DM scores if their family practice had a designated lead doctor for diabetes [10]. Mahdavi et al. (2021) have conducted a study to identify associations between health services operational factors and health experience for patients with type 2 diabetes in Iran. They concluded that continuity of care should be valued as an important structural element of an experience-based service delivery module for DM patients [49]. Also among the dimensions of COC, the relational continuity has een studied and focused in the most studies [50]. Chang et al. (2018) have examined the associations between COC, hospitalization and end-stage renal disease in DM patients in a cohort study in Taiwan. They have reported that COC is strongly correlated with end-stage renal disease and subsequent hospitalization among patients with DM. This study, suggested that when DM patients keep visiting the same physician for managing their disease, the progression of renal disease can be controlled [15]. Chen et al. (2022) in a cross-sectional study have studied the relationship between COC and self-management of patients with type 2 DM. They have showed that the continuity of care could explain more that 20% of total variance in self-management skill of patients that in return helps the management of the disease [51]. In another study, Hsieh et al. (2020), have examined the COC and its correlation with quality of life among 157 type 2 diabetes mellitus (T2DM) patients in Taiwan. In this study the average patient continuity of care questionnaire has reported as 50.11/60 and the mean score for each domain ranging from 4.12 to 4.21. Also, the authors have reported that the relational continuity with medical providers is among the most important predictors that effectively explore the patients’ quality of life [17]. Pandhi et al. (2006) in their study reviewed the literature regarding patients’ perceptions of interpersonal continuity of care to determine which patients value interpersonal continuity and in what context. Their study showed that Interpersonal continuity of care is important to a majority of patients, particularly those from vulnerable groups [52].

Multivariable regression analysis to investigate predictors of COC mean scores showed that only the age for the diabetes patients and insurance status for the hypertension patients act as the predictor of COC mean scores. As same as our study, Shin et al. (2021), have used multivariate regression analysis in their cross-sectional study to determine the related factors of COC in a sample diabetic patients at Seoul, Korea. They have showed that 3 categories of factors i.e. patients’ factors; clinic workforce factors and geographical factors including age, residential area, presence of disability, physician specialty, clinic location, hospitalization facility and distance between the patient location and their primary care clinic have significant correlations with the COC scores [8]. Stafford et al. (2023) in another study on the 381,474 patients suffering different conditions have reported that ethnic minority identity and socioeconomic deprivation have additive associations with lower continuity of care. They concluded that these results contribute to inequalities in experience and outcomes of patients [11]. Leniz and Gulliford (2019) in a national survey in Chile to investigate the continuity of care and delivery of diabetes and hypertensive care among regular users of primary care services have reported that COC is positively associated with age > 65 years, being female, retired, obese, high cardiovascular risk and widowed while negatively associated with educational level, smoking and physical activity [33]. Alazri et al. (2006), in another study in UK have showed that patients’ perceptions of continuity are influenced by several factors including a personal relationship between themselves and their healthcare professional, their own beliefs and behaviors, presence of diabetes, and the systems and structures of general practices [53].

Our findings documented a serious disruption in the continuity of care for chronic patients during the pandemic. This situation can intensify background diseases and impose a variety of negative outcomes to patients, families, and the whole community. Such negative outcomes could lead to a significant increase in the burden of chronic disease and health equalities globally [22]. It is obvious that this access reduction can lead to late diagnosis, inefficient treatment, and incremental trend of disease development [54]. Fekadu et al. (2021) in a review on the countries with limited resources have clarified that COVID-19 not only can led to higher risks among the elderly population and those with background chronic diseases but also it can act as a serious boundary against diagnosis, treatment, and prevention of chronic diseases [55]. Singh et al. (2022), also in a cross-sectional study conducted in several Asian countries, indicate that COVID-19 pandemic caused a poorer economic status, intensified the challenges in access to health care and worsened the chronic diseases symptoms and outcomes. Such side-effects are more tangible among rural and remote areas that can make the issue a global priority [56]. Yoon et al. (2022), in their qualitative study among micro, mezzo and macro level experts and policy makers in Singapore have demonstrated that COVID-19 made a variety of challenges in managing chronic diseases. These challenges are mainly because of limitations in direct relationships between the patients and health care providers, uncertainty in diagnostic clinical decisions due to protocol revisions and lack of access to pathological laboratories [40]. Therefore, applied interventions based on the local circumstances of each health system could be helpful here.

Serious attention to flexible and sustainable investments on global public health has been mentioned as a helpful strategy for this situation [31]. Recommendations can be focused on designing a model of care for chronic diseases which can assure the continuity of care during health disasters. Such a model should be able to facilitate the identification of high-risk patients and development of an applied plan for organizing chronic care and applying digital health for patient support. Community engagement for redesigning the health systems and attention to the community support, preparedness and surveillance of the health system also would be among considerable recommendations for health policy makers to ensure the COC for chronic patients during health crisis. Also, developing the tele-medicine infrastructures can be considered as an appropriate and applicable solution to manage the chronic non-communicable diseases. Parkinson et al. (2022) in their study have revealed the necessity for primary health care capacity building and continuity and comprehensiveness of chronic treatments now and in future. According to their findings, developing digital health, redesigning available models of care and applied research for identifications and interventions of the best solutions will be helpful [42]. Other approaches like strategic developing of Public-Private-Partnership for managing the chronic diseases and equal considerations to all the social and psychological aspects of non-communicable patients especially among the vulnerable groups should be taken into consideration.

### Strengths and limitations

Among the strengths of this study, we can mention that, firstly, the present article not only analyzed the registered data in health centers but also investigated the viewpoints of the patients about continuity of services during COVID-19. Secondly, this study has generated a new knowledge related to a developing country with restricted resources that could present applied managerial implications.

Considering the limitations, applying a longitudinal approach can present a more comprehensive picture of the outcomes of COVID-19 pandemic for patients with chronic conditions that is strongly recommended to be considered in future studies. Documentation of the health care providers isn`t also included in the present analysis. These results accompanied with investigating the changes in patients` clinical conditions during the pandemic can be mentioned for future studies.

## Conclusion

COVID-19 pandemic has affected the continuity of care among the patients with chronic condition. These side effects can be mainly due to the traffic restrictions, limited access to health care providers, concentration of the health systems on preventing and managing the pandemic and burnout prevalence among health care workers especially in those countries with restricted resources. Such a decline can worsen the condition of the patients with chronic problems in the long term and at the same time, impose irrecoverable challenges to the communities and health systems. Seeking the local and applied interventions to increase the health systems` resilience during health disasters should be considered as a new agenda by health policy makers. Developing the modern digital health technologies along with building the capacity for primary health care, developing inter-sectorial relationships, allocating sustainable resources and enabling the patients should also take into consideration for future policy making.

## Data Availability

Data available on request from the corresponding author (gh.a.tehrani@gmail.com).

## Abbreviations

DM: Diabetes Mellitus
HT: Hypertension
NCDs: Non-communicable Diseases
COC: Continuity of Care
PCCQ: Patient Continuity of Care Questionnaire
LC: Longitudinal Continuity
FC: Flexible continuity
RC: Relational Continuity
TCB: Team and Cross-boundary Continuity
ECC-DM: Experienced Continuity of Care for Diabetes Mellitus
T2DM: Type 2 Diabetes Mellitus
